# A prospective cohort study on cognitive and psychological outcomes in COVID-19 ICU survivors at 3 months of follow up

**DOI:** 10.3389/fmed.2024.1288761

**Published:** 2024-07-31

**Authors:** Merlin Thomas, Mansoor Hameed, Mousa Hussein, Saibu George, M. R. Rajalekshmi, Jaweria Akram, Rohit Sharma, Aisha Hussain O. Al Adab, Mushtaq Ahmad, Rajvir Singh, Tasleem Raza

**Affiliations:** ^1^Pulmonary Division, Department of Chest, Hamad General Hospital, Doha, Qatar; ^2^Department of Clinical Medicine, Weill Cornell Medicine, Doha, Qatar; ^3^Department of Medical Intensive Care, Hamad General Hospital, Doha, Qatar; ^4^Department of Clinical Medicine, Qatar University, Doha, Qatar; ^5^Department of Medical Research Centre, Hamad Medical Corporation, Doha, Qatar; ^6^Department of Internal Medicine, Geisinger Health System, Danville, PA, United States; ^7^Medical Research Centre, Hamad Medical Corporation, Doha, Qatar

**Keywords:** COVID-19, depression, anxiety, stress, cognitive function, psychological function

## Abstract

**Objective:**

The Outcomes – Short and Long term in ICU patient with COVID-19 “OUTSTRIP COVID-19” study was initiated to assess overall mortality, physical and psychiatric co-morbidities, reduction in lung function, and the ability to return to work post-ICU discharge with a follow-up period of 2 years in COVID-19 patients admitted to ICUs in Qatar. This paper focuses on the prevalence of cognitive impairment, depression, anxiety, and stress at baseline and 3 months after ICU discharge.

**Methods:**

This prospective cohort study included 100 ICU survivors reviewed at baseline within 7 weeks of ICU discharge, with a 3-month follow-up. Demographics, clinical characteristics, and relevant medical history were collected at baseline. Cognitive outcomes were assessed using the Montreal Cognitive Assessment-Basic (MoCA-B) tool, while psychological outcomes were evaluated using the Depression Anxiety and Stress Scale-21 (DASS-21).

**Results:**

At baseline, 72% of ICU survivors exhibited mild cognitive impairment, which significantly improved to 56% at 3 months. However, severe cognitive impairment persisted in 20% of survivors at 3 months.

For psychological outcomes, the mean depression score remained below 9 (5.64 ± 6.90) at both time points, with no significant change. At baseline, 25% of survivors had clinical depression, which reduced to 16% at 3 months.

The mean anxiety score at baseline (9.35 ± 8.50) significantly decreased to 6.51 ± 7.74 (p = 0.002) at 3 months. Anxiety was not reported by 48% of survivors at baseline and this increased to 66% at 3 months. Severe to extremely severe anxiety decreased from 19% to 12% during the same period.

The mean stress score at baseline (8.34 ± 8.07) did not significantly change at 3 months. At baseline, 18% experienced stress, which decreased to 12% at 3 months, with 5.3% facing severe to extremely severe stress.

**Conclusion:**

COVID-19 ICU survivors experience significant cognitive impairment, anxiety, and stress. While cognitive impairment and anxiety showed improvements at 3 months, depression and stress remained unchanged. These outcomes strongly emphasize the requirement for thorough post-ICU care and comprehensive mental health assistance for individuals recovering from COVID-19. Customized interventions and additional research endeavors are crucial to effectively manage the cognitive and psychological consequences faced by these patients. The exploration of telemonitoring and innovative approaches can offer avenues to enhance the overall quality of life for survivors. Further investigation should encompass extended timeframes to analyze prolonged effects and consider the broader socioeconomic impact.

## Introduction

Significant advancements in critical care medicine have yielded a growing cohort of Intensive Care Unit (ICU) survivors, giving rise to a spectrum of short and long-term health and socioeconomic effects (1). These consequences extend beyond acute respiratory distress syndrome (ARDS) survivors to other ICU treated diseases as well (2). Within this population, mental health repercussions, ranging from depression and anxiety to post-traumatic stress disorder (PTSD), have been reported in varying percentages, spanning from 8 to 57% (3, 4). Concurrently, cognitive impairment, persisting for months to years, affects a considerable proportion, with prevalence rates ranging from 30 to 80% (1, 5). The onset of the pandemic caused by the severe acute respiratory syndrome coronavirus 2 (SARS-CoV-2) has shed further light on the lingering neuropsychiatric and cognitive effects that may persist after recovery from intensive care or a critical illness (3). Long-lasting neuropsychiatric and cognitive effects, such as mental fatigue, PTSD, depression, and anxiety (4–23%), can manifest 2 to 12 months following COVID-19 (6, 7).

COVID-19 ICU survivors exhibit distinct features due to the specific pathophysiology of COVID-19. Factors such as inflammation, endothelial dysfunction, and microthrombi contribute to this uniqueness (8). Additionally, the severity of illness in COVID-19 patients often surpasses that of non-COVID-19 ICU survivors, resulting in divergent recovery trajectories.

Long-term outcomes are shaped by a combination of both ICU-related factors and COVID-19-specific elements. Researchers continue to investigate the intricate interplay between COVID-19 and its impact on ICU survivors.

Cognitive and psychological outcomes are influenced by various factors, such as genetics, social and cultural aspects, prior psychological disorders, occupational and financial stability, among others (8). As a result, findings from a particular study may not be applicable to populations in different geographical areas. Gulf Cooperation Council countries, including Qatar, have distinct population demographics. Qatar’s population predominantly comprises expatriates from over a hundred nationalities, representing a diverse tapestry of cultural and ethnic backgrounds, with the majority being male and below 60 years of age (9). Furthermore, Qatar boasts one of the lowest recorded COVID-19 mortality rates, with 682 deaths out of a total of 0.45 million cases as of September 2022 (10).

However, the COVID-19 pandemic has presented unique stressors for the majority expatriate population in Qatar. These stressors include fear of serious illness and death without close family support, financial fragility due to income loss, increased isolation due to social distancing rules and heightened travel restrictions limiting face-to-face interactions with families and loved ones. Our study, Outcomes−Short and Long term in ICU patient with COVID-19 “OUTSTRIP COVID-19” evaluated the patients’ mortality, co-morbidities, lung function, physical and psychiatric co-morbidities, and work capacity after ICU discharge.

This paper from OUTSTRIP COVID-19, seeks to examine the cognitive and psychological outcomes in COVID-19 patients admitted to ICUs in Qatar. The research aims to assess the prevalence of cognitive impairment, depression, anxiety, and stress at baseline and 3 months after ICU discharge, with a focus on the short-term follow-up.

## Materials and methods

### Study design, settings and population

We conducted a single-center prospective observational cohort study at Hamad Medical Corporation (HMC), a tertiary healthcare facility in the State of Qatar. HMC provided treatment for all patients with severe and critical COVID-19 in Qatar. The study received approval from the local Institutional Review Board (IRB) with the reference number MRC-05-073

The study was conducted in compliance with the Declaration of Helsinki. One hundred patients with COVID-19 who required ICU care were enrolled in the study within seven weeks of discharge from ICU (from August 10th-2020-May 5th, 2021). Patients over the age of 18 who were SARS CoV-2 positive based on Nucleic Acid Amplification Testing by reverse transcriptase PCR test detecting viral RNA in any respiratory secretion, admitted to ICU because of severe/critical COVID-19 illness, and able to provide valid informed consent before discharge or at first appointment in COVID chest clinics within 7 weeks of ICU discharge were eligible. Anyone with a suspected acute brain lesion that could cause global impairment of consciousness or cognition, such as traumatic brain injury, stroke, intracranial hemorrhage, or hypoxic brain injury, a preexisting neuro-psychological condition, moderate-to-severe COPD, asthma, cystic fibrosis, or parenchymal lung disease, i.e., interstitial lung disease, was excluded. Severe or Critical disease was defined as a positive COVID-19 test along with any of the following: dyspnea (respiratory rate ≥ 30 breath/min), hypoxia (SpO_2_ ≤ 93% on room air), radiological changes affecting ≥ 50% of the lung, or severe disease complications such as respiratory failure, the need for mechanical ventilation, septic shock, or non-respiratory organ failure.

The second follow-up was conducted at 3 months after their discharge from the ICU. Detailed information about the inclusion and exclusion criteria, as well as study definitions, can be found in a previous paper published by the same study group (11).

### Study variables

Baseline data and demographic information were collected when patients were admitted to the Intensive Care Unit (ICU) using a case record form. This comprehensive data encompassed various variables, including patient characteristics such as age and nationality, body mass index, results of blood tests and radiological examinations, presence of other medical conditions, length of hospital and ICU stay, total days on ventilator support, Acute Physiology and Chronic Health Evaluation (APACHE) score after 24 h, oxygen saturation index, and the ratio of arterial oxygen tension to the fraction of inspired oxygen (PaO2/FiO2 ratio).

To assess cognitive and psychological outcomes, two physicians and one research assistant utilized the following tools:

(a) Montreal Cognitive Assessment-Basic (MoCA-B): A validated screening tool used to evaluate mild cognitive impairment in populations with varying literacy and low education levels (12). The MoCA-B scores range from 0 to 30 points, encompassing visual perception, executive functioning, language, attention, memory, and orientation. The MoCA-B is available in English, Hindi, Bengali, and Arabic (13). A score of < 24/30 (with 81% sensitivity and 86% specificity) was considered the cut-off for mild cognitive impairment (12). The validity and reliability of the Montreal Cognitive Assessment-Basic (MoCA-B) have been investigated in both the Middle East and North Africa (MENA) region and the Indian population. This is particularly relevant as the Indian population constitutes a major demographic group in Qatar (14, 15).
(b) Depression Anxiety and Stress Scale-21 (DASS-21): This validated questionnaire consists of 21 items, with 7 items each measuring depression, anxiety, and stress (16). Scores obtained classify depression, anxiety, and stress levels as mild, moderate, severe, or extremely severe. The DASS-21 has been widely used in multiple studies to assess psychological outcomes. The Depression Anxiety and Stress Scale-21 (DASS-21) has been validated and proven to be reliable in the Middle Eastern population (17).

### Statistical analysis

Data was managed and analyzed using Microsoft Excel and SPSS version 28 (IBM Corp. Released 2021. IBM SPSS Statistics for Windows, Version 28.0. Armonk, NY: IBM Corp). Descriptive statistics using mean (SD) and frequency (%) were used to describe the data. Mean and standard deviations were calculated for interval variables, while frequency distribution with percentages was computed for categorical variables in the study. Paired student *t*-tests were utilized to assess mean significant differences in variables, including MOCA score, MOCA time, DASS depression, DASS stress, and DASS anxiety scores at baseline and three months. Chi-square tests (McNemar) were employed for all categorical variables. A *p*-value of 0.05 (two-tailed) was considered as the level of statistical significance. To account for important confounders such as age and gender, a multivariate mixed-method model with repeated measures ANOVA and post-hoc analysis using Bonferroni was conducted to examine the effect of anxiety scores from baseline to 3 months.

## Results

**FIGURE 1:**
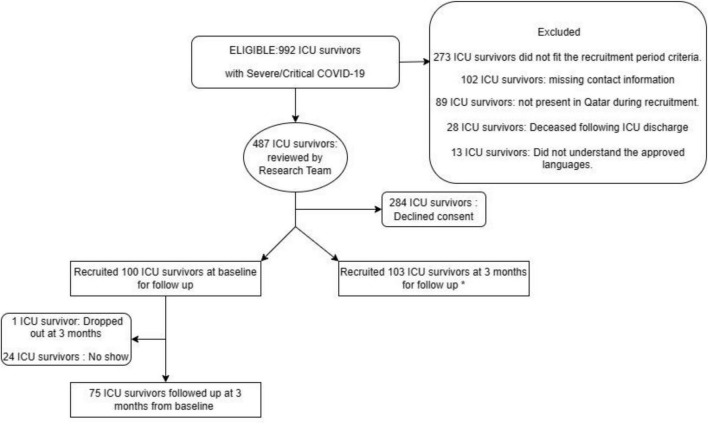
Flow chart of patient selection and follow up at 3 months. *This paper has reported data of patients recruited at baseline and followed up at 3 months. No Show = Patients who did not attend follow up appointments at 3 months.

A total of 100 ICU survivors were reviewed at baseline within 7 weeks of discharge from the ICU, among whom 24 survivors did not attend visit 2 at 3 months due to logistical reasons related to work, transport, and travel. One participant dropped out of the study as indicated in Figure 1.

Demographics and clinical characteristics of the cohort of ICU survivors are presented in Table 1. In accordance with the inclusion-exclusion criteria, there were no known cognitive, psychiatric, or psychological illnesses prior to ICU admission for COVID-19. The participants’ mean age was 47.87 ± 8.4 years, with the majority being males (82%) and nonsmokers (94%). Major co-morbidities included diabetes mellitus (56%) and hypertension (44%).

**TABLE 1:** Baseline Characteristics of study participants.

Baseline variables	Number of patients *N* (%)
Gender (male)	82 (82.0)
Age, years, mean ± SD	47.87 ± 8.4
BMI, mean ± SD	30.5849 ± 5.9
Nationality (Qatari)	2 (2.0)
Smoking	6 (6.0)
**Comorbidities**	
Diabetes Mellitus	56 (56.0)
Hypertension	44 (44.0)
Chronic cardiac Disease	1 (1.0)
Chronic Lung disease	8 (8.0)
Cancer	1 (1.0)
Immunocompromised	1 (1.0)
**Treatment Received during hospital stay**	
Corticosteroids	99 (99.0)
Remdesivir	81 (81.0)
Tocilizumab	37 (37.0)
Vasopressors	17 (17.0)
Sedatives	20 (20.0)
Convalescent plasma therapy	43 (43.0)
Blood Transfusion	6 (6.0)
Narcotics, *n* (%), Days (mean ± SD)	21 (21), 6.9 ± 3.8
Benzodiazepine, *n* (%), Days (mean ± SD	21 (21), 5.04 ± 3.06
Neuroleptics, *n* (%), Days (mean ± SD	9 (9), 6.4 ± 4.5
**Oxygen support**	
Face mask/Non rebreathing Mask	8 (8%)
Non-invasive ventilator	72 (72.0)
Mechanical ventilation	20 (20.0)
Prone Positioning	27 (27.0)
**Length of stay**	
Intensive Care unit, days	10.78 ± 12.3
Total hospital stay	19.72 ± 17.547

During their hospital course, 20% of the patients required invasive mechanical ventilation, while the majority 80% were managed with noninvasive ventilation or supplemental oxygen therapy. The mean duration of stay in the ICU was 10.7 ± 12.3 days, while the total duration of hospital stay was 19.7 ± 17.5 days. Among the participants, 43% received convalescent plasma, and 6% required blood transfusion. In terms of sedatives and analgesics, narcotics were administered to 21 patients for an average of 6.9 ± 3.8 days, benzodiazepines to 21% of patients for 5.04 ± 3.06 days, and neuroleptics to 9% of patients for 6.4 ± 4.5 days. Delirium was observed in 9% of the ICU survivors.

### Results on depression, anxiety and stress assessment by DASS-21

Depression, DASS-D: The mean depression score remained within the normal scale (below 9) at 5.64 ± 6.90, with no significant improvement noted at 3 months, as shown in Table 2. However, at baseline, 25% of the 100 survivors exhibited clinical depression ranging from mild to extremely severe. This percentage reduced to 16% (12 survivors) at 3 months, as indicated in Table 3.

**TABLE 2:** Montreal Cognitive assessment−basic (MoCA-B) and Depression Anxiety and Stress Scale-21 (DASS-21) at baseline and 3 months follow up.

Parameter	Baseline, *n* (%) *N* = 100	3-month, *n* (%) *N* = 75	*P*-value
**MOCA−B**			
Mean Value, mean ± sd	26.5 ± 2.6	27.4 ± 2.02	< 0.001
Light cognitive impairment (≥ 26)	72 (72.0)	56 (74.7)	
Proven cognitive (< 26)	28 (28.0)	20 (26.7)	
Time taken to complete, minutes, mean ± sd	8.9 ± 2.0	7.3 ± 1.7	< 0.001
**DASS-21**			
Depression, mean ± sd	5.31 ± 6.60	4.37 ± 6.80	0.194
Anxiety, mean ± sd	9.13 ± 8.53	6.51 ± 7.74	0.002
Stress, (mean ± sd	8.27 ± 8.	7.15 ± 7.50	0.154

**TABLE 3:** Depression (DASS 21-D) Anxiety (DASS 21-A) and Stress (DASS 21-S) at baseline and 3 months follow up.

DASS 21-D	Baseline *N* = 100	3month *N* = 75	*P* value	DASS 21-A	Baseline *N* = 100	3month *N* = 75	*P*-value	DASS 21-S	Baseline *N* = 100	3month *N* = 75	*P*-value
Normal (0–9)	75 (75.0)	63 (84.0)	0.29	Normal (0–7)	48 (48.0)	50 (67.0)	0.11	Normal (0–14)	82 (82.0)	66 (88.0)	0.05
Mild (10–13)	11 (11.0)	5 (6.7)		Mild (8–9)	13 (13.0)	7 (9.3)		Mild (15–18)	9 (9.0)	4 (0.0)	
Moderate (14–20)	9 (9.0)	5 (6.7)		Moderate (10–14)	20 (20.0)	8 (10.7)		Moderate (19–25)	4(4.0)	1 (1.30)	
Severe (21–27)	4(4.0)	1(1.3)		Severe (15–19)	7 (7.0)	3 (4.0)		Severe (26–33)	4 (4.0)	3 (4.03.0)	
Extremely severe (28++)	1 (1.0)	1 (1.3)		Extremely severe (20+)	12 (12.0)	7 (9.3)		Extremely severe (34+)	1 (1.0)	1 (1.30)	

Anxiety, DASS A: The mean anxiety score exceeded the normal value of 7, with a significant improvement from baseline to 3 months (9.35 ± 8.50 versus 6.51 ± 7.74; *p* = 0.002). At baseline, 48% of survivors did not report anxiety, and this percentage increased to 66% (50 survivors) at the 3-month follow-up. However, severe to extremely severe anxiety was noted in 19% of survivors at baseline and decreased to 12% (9 survivors) at 3 months.

Stress, DASS-S: The mean stress score at baseline was 8.34 ± 8.07, falling within the normal range of less than 14, with no significant improvement at 3 months. However, 18% of patients experienced some degree of stress at baseline, with 5% facing severe to extremely severe stress. At the 3-month follow-up, only 12% of the patients still experienced stress, and out of those, 5.3% had severe to extremely severe stress.

### Results on cognitive outcomes by MOCA-B

The majority of the patients (72%) exhibited mild cognitive impairment, which showed significant improvement of 1 point (*p* < 0.001) at 3 months, as indicated in Table 2. The time taken to complete the MOCA-B questionnaire also significantly decreased by 1.6 minutes (*p* < 0.001) at 3 months.

However, severe cognitive impairment observed in 28% of survivors at baseline persisted in 20% of survivors at 3 months, while 56% continued to have mild cognitive impairment at 3 months, compared to 72% of survivors at baseline.

### Interaction between anxiety and age

Further correlation with invasive mechanical ventilation did not show any significant impact on the mean changes in MOCA-B and DASS-21 scores.

Within the analysis, the effect of anxiety (F: 4.84, *p* = 0.03) and the interaction between anxiety and age (F: 7.72, *p* = 0.007) were found to be significant, while the interaction between anxiety and sex was not significant (F: 0.24, *p* = 0.63). Regarding between subjects’ effect, age was not significant (F: 0.04, *p* = 0.84), but the effect size was significant between males and females (F: 5.45, *p* = 0.01), with estimated marginal means of 12.2 ± SE (1.92) and 6.8 ± SE (0.92), respectively.

However, parameters such as depression and stress were not found to be significant from baseline to 3 months, thus multivariate mixed-method model repeated measures ANOVA was not performed for these parameters.

## Discussion

Our investigation enhances the comprehension of the psychological well-being and cognitive status exhibited by individuals with COVID-19 who underwent admission to the ICU, revealing, that a substantial number of ICU patients exhibited cognitive impairment both at the baseline assessment and at the 3-month follow-up. This persistent cognitive impairment highlights the complex interplay between critical illness, COVID-19 infection, and potential neurological effects. Importantly, while the prevalence of depression and stress was reported by approximately one-quarter of the patients during the baseline assessment, more than half of the participants experienced notable levels of anxiety. This underscores the considerable psychological distress experienced by these patients during their ICU stay and its potential lingering impact. Although a trend towards improvement was observed in all parameters at 3 months, significant improvements were noted only in cognitive measurement and anxiety.

### Depression and anxiety

In a systematic review conducted by Renaud-Charest et al., it was demonstrated that the prevalence of depressive symptoms beyond 12 weeks following SARS-CoV-2 infection spanned from 11 to 28% (16). In line with these findings, our study echoed similar results, reporting depression symptoms in 16% of our cohort at the 12-week mark. The literature offers diverse insights into the factors linked with depression and anxiety, yielding contrasting outcomes. Some studies point to sex, previous psychiatric history, psychopathology at the one-month follow-up, and acute-phase systemic inflammation as potential contributors, while age emerges as a tentative factor and the severity of acute COVID-19 appears non-contributory.

Mazza et al. highlighted a connection between baseline systemic inflammation severity and depressive symptoms, with age showing no significant correlation (18, 19). Conversely, Morin et al. (20) identified age over 75 as a significant risk factor for depression (21). Interestingly, the severity of acute COVID-19, encompassing symptomatology and treatment intensity, including intubation, did not exert influence over the occurrence of depressive symptoms, as indicated by some studies integrated into Renaud-Charest et al.’s systematic review. Similarly, the long-term rates of depressive symptoms did not show significant elevation in hospitalizations for COVID-19 with neurological complications compared to those without such complications.

As our study’s trajectory extends to the 3-month mark, a significant improvement in anxiety was evident; however, a notable 12% (comprising 9 survivors) continued to experience severe to extremely severe anxiety. Notably, Zhang et al. (22) presented a different facet, revealing no statistically significant disparity in the prevalence of anxiety among COVID-19-infected individuals, those in quarantine, and the general population (20). Yuan et al. (23), in their meticulous systematic review and meta-analysis, unveiled a pertinent trend: the prevalence of depression, anxiety, and insomnia witnessed an uptick during the COVID-19 epidemic across various demographic subsets, spanning the public, healthcare professionals, university students, older adults, infected patients, survivors, and pregnant women. Intriguingly, while university students reported the highest pooled prevalence of depression, survivors of COVID-19 exhibited the lowest prevalence. Within this tapestry, anxiety garnered a high prevalence rate among pregnant women, while older adults reported the lowest levels. Our findings reveal a significant interaction between anxiety and age (F: 7.72, *p* = 0.007), suggesting that age modifies how anxiety affects cognitive and emotional outcomes in post-ICU COVID-19 patients. The influence of age on psychological recovery has also been noted in other studies, with older adults often exhibiting greater resilience in psychological outcomes post-critical illness and severe COVID-19 (20, 24). However, the trajectory of recovery can vary, and certain age groups may experience prolonged anxiety symptoms, potentially due to pre-existing conditions, the severity of the illness, or social factors such as isolation (25).

Interestingly, our analysis showed a significant effect size between males and females concerning the impact of anxiety on outcomes (F: 5.45, *p* = 0.01). This observation is crucial, as it aligns with literature suggesting that sex differences may influence the prevalence and manifestation of psychological symptoms after traumatic health events. Females often experience longer and more severe post-COVID-19 syndrome, with a higher incidence of depression compared to males (26). Additionally, in a Swedish cohort followed for one year after ICU admission with COVID-19, female sex was a predictor of depression (27). Our results indicate that males might also be significantly impacted, potentially due to socio-cultural roles or differences in coping mechanisms (28). Female gender could be emerging as a risk factor for depression likely due to other factors such as pre-existing depression, cultural norms, gender-based biases and expectations, and hormonal changes in women, particularly those in the perimenopausal age group (27). However, we have a relatively young cohort of mixed nationalities and predominantly male participants, resulting in unique findings.

The overarching prevalence of mental health concerns during the COVID-19 epidemic exhibited variability across different countries (29). Within this complex framework, the extent to which the amplified media coverage of COVID-19 shapes the articulation of symptoms remains enigmatic, holding the potential to temper or intensify symptom severity and overall quality of life reporting. A realm of speculation also emerges, pondering whether COVID-19 survivors might have benefited from augmented clinical and social support, including concentrated attention on post-discharge education, thereby potentially leading to a diminished prevalence of anxiety and depression. This could potentially be true for our cohort as Qatar had launched a robust mental health services program targeting the public, covid-19 patients in quarantine and inpatients from admission to discharge and follow up (22).

Indeed, the socioeconomic ripple effects of the pandemic could potentially shape the frequency and severity of depression, necessitating a more in-depth investigation. Amidst the direct specter of the virus, the pandemic has catalyzed a cascade of uncertainties, spanning employment, housing, education disruptions due to school closures, and the amplification of social isolation, yet the complete complexion of these dynamics remains to be meticulously elucidated.

### Cognitive impairment

Most of our patients (72%) displayed mild cognitive impairment, while 28% presented with severe impairment; however, both groups demonstrated significant enhancement in cognitive function at the 3-month follow-up. Notably, participants exhibited a reduced completion time for the questionnaire compared to baseline, potentially attributed to substantial advancements in visual memory, processing speed, and attention over the observation period (23). Ceban et al. (30), in their metanalysis, unveiled that a proportion exceeding one-fifth of individuals exhibited cognitive impairment extending beyond 12 weeks post-confirmed COVID-19 diagnosis. Strikingly, similar incidences of cognitive impairment and fatigue were observed across hospitalized and non-hospitalized cohorts. Furthermore, their synthesis alludes to a distinctive pattern whereby fatigue and cognitive impairment endure and may even exacerbate over time in vulnerable individuals, substantiated by similar proportions of afflicted individuals across both < 6 and > 6 month follow-ups (31).

Conversely, certain longitudinal inquiries have probed the temporal dimensions of cognitive dysfunction and indicated its potential non-permanence. A study encompassing 78 COVID-19 patients in Ecuador revealed that though MOCA scores at the 6-month follow-up were notably lower among COVID-19 patients in contrast to those without, no significant difference was discerned at the 18-month mark (32). A case-control study aligned with this narrative, as it reported the absence of consequential cognitive impairment after the passage of 6 to 9 months, suggesting the potential for recovery over time (30). Similarly, a prospective longitudinal examination of COVID-19 cases in the USA, following a year of hospitalization, exhibited pronounced improvements in telephonic MOCA scores (56% improvement, median 1 point, *p* = 0.002) and Neuro-Quality of Life anxiety scores (45% improvement, *p* = 0.003) between the 6 to 12-month interval (33, 34). Through our extended 24-month longitudinal investigation, OUTSTRIP COVID study, we aim to hopefully offer further insights into the enduring or transient nature of cognitive repercussions stemming from COVID-19.

The pathophysiological substrates that facilitate the emergence or exacerbation of persistent fatigue and cognitive impairment after SARS-CoV-2 infection are intricate and multifaceted. A spectrum of factors have been discerned, encompassing direct viral encephalitis, neuroinflammation, hypoxia, cerebrovascular perturbations, microvascular injury within the cerebral domain, endothelial dysfunction, autoimmunity, latent viral resurgence, multi-organ involvement, and even autonomic nervous system aberrations (35, 36). Limited evidentiary strands allude to a conceivable association linking escalated pro-inflammatory markers with fatigue and cognitive impairment. Subsequent research endeavors should take the lead in unraveling the underlying mechanisms, crafting standardized diagnostic criteria, and forging therapeutic modalities aimed at averting and ameliorating fatigue and cognitive impairment within the post-COVID-19 domain.

### Strengths and limitations

Strengths of our study include its longitudinal design, which allowed for the collection of detailed and accurate information to minimize recall bias. Thorough and detailed measurements of outcome variables and random selection of participants ensured a representative sample size.

However, our study also has some limitations, including a small sample size, inability to analyze individual variables as predictors of outcomes, and limited generalizability due to the unique population mix. The heterogeneity of ethnic and cultural groups further limits generalizability to specific populations. The absence of a non-COVID control group also hinders the evaluation of the isolated effect of COVID on outcomes. Additionally, regarding cognitive assessment, we did not measure individual domain scores of the MOCA-B questionnaire, restricting the comparison of these domain scores and the measurement of improvement. In future studies aiming to parse out the distinct impacts of COVID-19 and ICU care, researchers should consider including several control groups to enhance the specificity of their findings. One recommended approach is to recruit a control group composed of patients who have been hospitalized for COVID-19 but did not require ICU admission. This group would help to isolate the effects of the virus itself from the intensive treatments associated with ICU care. Additionally, a second control group could consist of ICU patients who were admitted for reasons other than COVID-19. This would allow researchers to examine the unique psychological and physiological impacts of ICU care, irrespective of COVID-19 infection. To further refine the analysis, a third control group of individuals from the general population, matched for relevant demographic characteristics such as age, sex, and pre-existing health conditions, could also be included. This group would provide a baseline, helping to assess the impact of hospitalization and critical care against the general health outcomes experienced by the wider community. Utilizing these distinct control groups will enable researchers to conduct a more nuanced analysis, yielding insights that are critical for developing tailored interventions designed to mitigate the long-term effects of both COVID-19 and ICU admissions.

## Conclusion

In conclusion, our study provides critical insights into the psychological and cognitive sequelae experienced by ICU survivors of COVID-19 at the three-month post-discharge mark. The findings underscore the significant prevalence of anxiety, depression, and cognitive impairments, which have profound implications for the assessment and treatment of this patient population.

### Assessment impact

The use of standardized tools like the DASS-21 and MOCA-B in our study emphasizes the importance of regular and systematic evaluation of mental health and cognitive function in ICU survivors. These assessments should be integrated into routine post-ICU care protocols to identify individuals at risk of prolonged psychological distress and cognitive deficits.

### Treatment impact

The persistence of anxiety and cognitive impairments at three months indicates that a substantial proportion of ICU survivors may benefit from targeted interventions. This could include psychological support, cognitive rehabilitation, and pharmacological management tailored to individual patient needs. Our study advocates for the early initiation of such interventions to mitigate the long-term consequences of ICU stay on mental health.

Furthermore, the study’s findings highlight the necessity for a multidisciplinary approach to post-ICU care. This approach should involve primary care physicians, mental health professionals, and rehabilitation specialists working collaboratively to address the complex needs of COVID-19 ICU survivors.

By shedding light on the specific challenges faced by ICU survivors, our study contributes to the ongoing discourse on optimizing post-ICU recovery pathways. It calls for the development of personalized, population-focused strategies that consider the unique demographic and cultural characteristics of patients, which is particularly pertinent in diverse societies.

Ultimately, the present study serves as a clarion call for healthcare systems to bolster their post-ICU support structures, ensuring that survivors of critical illness receive the comprehensive care necessary to improve their quality of life in the long term. We hope to build on the findings of this study by publishing further data on the 2-year post-ICU survival soon, which will provide additional insights into the long-term recovery trajectories of these patients.

## Data availability statement

The original contributions presented in the study are included in the article/supplementary materials, further inquiries can be directed to the corresponding author.

## Ethics statement

The studies involving humans were approved by the Medical Research Center in HMC. The studies were conducted in accordance with the local legislation and institutional requirements. The participants provided their written informed consent to participate in this study.

## Author contributions

MT: Conceptualization, Data curation, Formal analysis, Investigation, Methodology, Supervision, Writing−original draft, Writing−review and editing. MaH: Conceptualization, Formal analysis, Investigation, Writing−review and editing. MoH: Conceptualization, Investigation, Methodology, Supervision, Writing−original draft, Writing−review and editing. SG: Data curation, Investigation, Methodology, Writing−review and editing. MR: Data curation, Investigation, Methodology, Supervision, Writing−original draft. JA: Data curation, Investigation, Writing−review and editing. RS: Data curation, Investigation, Validation, Writing−original draft. AA: Data curation, Formal analysis, Investigation, Methodology, Supervision, Writing−review and editing. MA: Formal analysis, Investigation, Methodology, Writing−original draft. RS: Conceptualization, Formal analysis, Methodology, Project administration, Software, Writing−original draft. TR: Conceptualization, Investigation, Methodology, Supervision, Validation, Visualization, Writing−original draft, Writing−review and editing.
