# Corrigendum: A prospective cohort study on cognitive and psychological outcomes in COVID-19 ICU survivors at 3 months of follow up

**DOI:** 10.3389/fmed.2025.1565509

**Published:** 2025-03-14

**Authors:** Merlin Thomas, Mansoor Hameed, Mousa Hussein, Saibu George, M. R. Rajalekshmi, Jaweria Akram, Rohit Sharma, Aisha Hussain O. Al Adab, Mushtaq Ahmad, Rajvir Singh, Tasleem Raza

**Affiliations:** ^1^Pulmonary Division, Department of Chest, Hamad General Hospital, Doha, Qatar; ^2^Department of Clinical Medicine, Weill Cornell Medicine, Doha, Qatar; ^3^Department of Medical Intensive Care, Hamad General Hospital, Doha, Qatar; ^4^Department of Clinical Medicine, Qatar University, Doha, Qatar; ^5^Department of Medical Research Centre, Hamad Medical Corporation, Doha, Qatar; ^6^Department of Internal Medicine, Geisinger Health System, Danville, PA, United States; ^7^Medical Research Centre, Hamad Medical Corporation, Doha, Qatar

**Keywords:** COVID-19, depression, anxiety, stress, cognitive function, psychological function

In the published article, there was an error. The incorrect funder was listed. A correction has been made to the **Funding** and **Acknowledgments**.

The correct Funding statement appears below.

